# Feasibility of acceptance and commitment therapy for repetitive negative thinking versus internet-based CBT: a pilot randomised controlled trial in Swedish primary care among patients with anxiety, depression and stress

**DOI:** 10.1186/s40814-026-01951-2

**Published:** 2026-10-31

**Authors:** Thor Bengtsson, Moa Wolff, Andreas Larsson, Peter Nymberg, Veronica Milos Nymberg

**Affiliations:** 1 Center for Primary Health Care Research, Department of Clinical Sciences Malmö, Lund University, Malmö, Sweden; 2 University Clinic Primary Care, Skåne University Hospital, Malmö, Region Skåne Sweden; 3 Department of Psychology and Social Work, Clinical Psychology, Mid Sweden University, Östersund, Sweden

**Keywords:** Acceptance and commitment therapy, Internet-based intervention, Cognitive behavioural therapy, Rumination, Cognitive, Depression, Anxiety, Primary health care, Pilot projects, Sweden

## Abstract

**Background:**

Repetitive negative thinking (RNT) is a transdiagnostic process relevant to common mental health problems in primary care. RNT-ACT is a brief acceptance and commitment therapy-based protocol targeting RNT in two sessions. This pilot study assessed the feasibility of RNT-ACT compared with therapist-supported internet-based cognitive behavioural therapy (iCBT) in Swedish primary care.

**Methods:**

Thirteen participants were randomised: 7 to RNT-ACT and 6 to iCBT. The primary feasibility outcomes were recruitment rate, intervention non-completion, and patient satisfaction measured with the Client Satisfaction Questionnaire (CSQ-8). Symptom and process measures were secondary outcomes. Secondary outcomes were assessed at baseline and at 1- and 3-month follow-ups. Analyses were descriptive and exploratory, based on available observations without imputation of missing data.

**Results:**

Thirteen of 20 eligible individuals were enrolled (65%), corresponding to 35% of all 37 individuals screened. All participants in the RNT-ACT group completed the intervention, whereas 4 of 6 participants in the iCBT group did not complete the intervention. Mean CSQ-8 scores were 27.8 (SD 3.7; *n* = 5) in the RNT-ACT group and 23.0 (SD 0.0; *n* = 2) in the iCBT group.

**Conclusions:**

The small sample size and high iCBT non-completion limit between-group comparisons. Further investigation of recruitment, treatment engagement, and patient and therapist experiences is needed before determining whether and how to proceed to a full-scale RCT.

**Trial registration:**

ClinicalTrials.gov NCT05946382 registered July 14, 2023.

**Supplementary Information:**

The online version contains supplementary material available at https://doi.org/10.1186/s40814-026-01951-2.

## Key messages regarding feasibility

- What uncertainties existed regarding feasibility prior to this study? This is the first study on the psychological protocol RNT-ACT in a Primary Care setting. Consequently, feasibility parameters such as recruitment, retention, and adherence were unknown. In addition, the evidence on using iCBT as treatment-as-usual in this context was limited, making the feasibility assessment of the control group equally important.
- What are the key feasibility findings? Recruitment among eligible individuals was acceptable; however, the overall recruitment rate was low. Attrition was particularly high in the iCBT group, and completing the primary follow-up proved challenging.
- What are the implications of the feasibility findings for the design of the main study? The findings indicate that recruitment, treatment engagement, assessment burden, and the timing of research follow-up require further investigation before considering a definitive trial. Qualitative investigation of patient and therapist experiences may help inform future recruitment procedures, study design, and potential adaptations of the intervention.

## Background

Most patients in Sweden with mental illness are treated in primary care [1]. The implementation of psychological interventions in routine care requires consideration of how treatments fit existing clinical structures and resources [2]. In primary care, accessibility and integration with other healthcare services are therefore important considerations [3, 4]. Internet-based cognitive behavioural therapy (iCBT) is a standard treatment across Swedish regions with symptom outcomes comparable to face-to-face therapy and a unique national registry for psychotherapy evaluation [5–7]. A systematic review found iCBT to be more time-efficient for therapists than traditional CBT [8]. However, routine digital management and integration with electronic health records add time, a factor rarely reported in the literature. Studies report varying acceptance and initiation rates for iCBT, with some patients preferring face-to-face alternatives; one study found that as many as 40.6% of primary care patients preferred other options [8, 9]. Although the differences are generally small, dropout rates tend to be lower for face-to-face therapy [7]. Overall, these findings indicate that engagement with psychological treatment can be challenging in routine care.

Transdiagnostic models target shared psychological processes across disorders, offering broader applicability than diagnosis-specific treatments [2]. Repetitive negative thinking (RNT) contributes to conditions relevant to primary care, including depression, anxiety, PTSD, insomnia, pain, and alcohol use [10]. Addressing RNT may improve outcomes for common mental disorders and related chronic conditions, including medication adherence [11].

Acceptance and commitment therapy (ACT) is a form of CBT incorporating a behavioural theory of verbal learning, relational frame theory [12]. ACT is applied across psychiatric and somatic conditions in different forms and settings, and the evidence base is growing [13]. The RNT-ACT protocol (acceptance and commitment therapy for repetitive negative thinking) targets worry and depressive rumination in two 60-min sessions, aiming to improve accessibility. In a Colombian randomised trial, a two-session RNT-ACT protocol showed large effects on depression and generalised anxiety symptoms compared with a waitlist control [14]. However, the feasibility of delivering and evaluating this specific two-session RNT-ACT protocol within routine Swedish primary care had not previously been investigated, including recruitment, treatment engagement, and research follow-up in this setting. RNT-ACT differs from broader ACT approaches by specifically targeting RNT using a functional contextual and relational frame theory (RFT)-based conceptualisation of worry and rumination as patterns of experiential avoidance that can maintain negative affect and interfere with valued action [15]. In 2020, four Swedish licensed psychologists, including the first author, translated the protocol while preserving the theoretical and functional structure [15]. The aim of this study was to assess the feasibility of the trial design in a clinical primary care setting, focusing on recruitment, intervention non-completion, and patient satisfaction. The secondary aim was to descriptively explore changes in psychiatric symptoms and process measures.

## Methods/design

### Setting and participants

#### Setting

The pilot study was conducted at an urban medium-sized Primary Health Care Centre (PHCC) in Skåne County, southern Sweden, serving approximately 7000 patients. The interventions were administered by the first author of this paper (TB), a licensed clinical psychologist and principal investigator of the study, hereafter referred to as the study psychologist.

#### Recruitment and screening

Patients were informed about the study via posters in the PHCC waiting room. Interested individuals could either contact the study psychologist directly or be referred by their primary care provider. They were informed about the enrolment procedure face-to-face or by phone and were able to ask questions. The study psychologist scheduled an enrolment session and sent the secondary outcome questionnaires digitally via secure communication. During the enrolment session, written informed consent was obtained, and the patient’s medical record was reviewed for recent blood tests. If no recent blood tests were available, the study psychologist consulted the patient’s physician regarding additional blood tests to rule out potential somatic causes as part of routine screening. The assessment session was primarily based on the clinical Mini International Neuropsychiatric Interview 7.0.1 [16–18], but also included information about the study, assessment of exhaustion using the Shirom-Melamed Burnout Measure-6 (SMBQ-6) [19], and randomisation to the treatment arms. The recruitment period was originally planned to run from January to April 2024.

#### Inclusion criteria

Participants were eligible if they were aged 18 years or older, had a DASS-21 total score >20, and were able to communicate in spoken and written Swedish. The DASS-21 >20 criterion was not intended as a diagnostic cut-off. The DASS-21 is a dimensional measure of symptom severity, and the 20/21 threshold was calculated using the Jacobson and Truax method based on Swedish DASS-21 data reported by Alfonsson et al. [20]. It was used as a symptom-related eligibility threshold for the present study.

#### Exclusion criteria

Participants were excluded if physical illness requiring further medical assessment was identified during routine screening. Routine blood tests included haemoglobin (Hb), thyroid-stimulating hormone (TSH), alanine aminotransferase (ALAT), aspartate aminotransferase (ASAT), serum creatinine, sodium (Na), and potassium (K). Participants receiving ongoing psychological or psychiatric treatment were also excluded.

Participants were also excluded if the M.I.N.I. 7.0.1 assessment indicated substance abuse, anorexia nervosa, a psychotic disorder, bipolar disorder, attention-deficit/hyperactivity disorder (ADHD), or antisocial personality disorder. Participants were excluded if the clinical assessment indicated a level of suicide risk that made participation in the study inappropriate. Suicidal thoughts alone did not constitute an exclusion criterion. No screened participant was excluded on the basis of suicide risk. Participants with a level of exhaustion considered incompatible with participation were excluded. Before September 5, 2024, this was based on clinical assessment informed by the Shirom-Melamed Burnout Measure-6 (SMBQ-6). Thereafter, the Karolinska Exhaustion Disorder Scale (KEDS) was used, with scores >29 indicating exclusion [21, 22].

#### Sample size

The target pilot sample size of 12 participants was selected pragmatically based on the planned 4-month recruitment period and the number of participants expected to be recruited during that period. The pilot was intended primarily to implement the trial procedures on a small scale and identify feasibility issues related to recruitment, intervention completion, patient satisfaction, and research follow-up before considering a definitive trial. It was also expected to provide preliminary locally generated outcome data relevant to planning a future definitive trial. The sample size was not intended to provide precise estimates of feasibility parameters or treatment effects, and no formal pilot-specific sample size calculation based on estimation precision was performed. This follows current guidance emphasising that pilot trial sample sizes should be aligned with the feasibility objectives and the information required to inform decisions about a future definitive trial [23].

### Study measures

#### Primary outcome measures (feasibility outcome measures)

The primary feasibility outcomes were recruitment rate, intervention non-completion, and patient satisfaction.

Recruitment rate was calculated as the proportion of enrolled participants among all screened individuals. Before study initiation, an overall recruitment rate of at least 50% and intervention completion of at least 80% were considered acceptable feasibility criteria. These thresholds were intended to inform whether modifications would be required before a definitive trial rather than to establish feasibility in isolation.

Intervention non-completion was defined as failure to complete the allocated intervention. Intervention completion of at least 80% was considered acceptable.

Patient satisfaction was assessed using the Client Satisfaction Questionnaire (CSQ-8), with scores ranging from 8 to 32 and higher scores indicating greater satisfaction. The reference mean of 24.16 (SD 4.94) was used as a descriptive benchmark for patient satisfaction [24].

#### Secondary outcome measures

The following secondary outcome measures were used:

- The Depression, Anxiety and Stress Scale-21 (DASS-21)
- Ruminative Responses Scale—Brooding and Reflection (RRS-BR)
- Swedish Acceptance and Action Questionnaire (SAAQ)
- Values Questionnaire (VQ)
- Cognitive Fusion Questionnaire—Swedish (CFQ-7)

Scores on validated self-report measures of depression, anxiety, stress, rumination, psychological flexibility, and cognitive fusion were collected, consistent with previous RNT-ACT studies [14]. Outcomes were assessed at baseline, at the 1-month post-intervention assessment (main follow-up), and at the 3-month follow-up. The M.I.N.I. was used as part of the eligibility assessment and to inform selection of the most appropriate iCBT program [16–18]. DASS-21 served as a global distress measure [20]. RRS-BR assessed rumination processes [25, 26]. SAAQ, VQ, and CFQ-7 assessed different aspects of psychological flexibility [27–30]. Details of the measures, including score ranges and psychometric information, are provided in Additional file 1.

#### Randomisation and blinding

A research assistant without patient involvement performed the randomisation. Participants were allocated 1:1 to RNT-ACT or iCBT using a computer-generated permuted block randomisation sequence with a fixed block size of 12, generated using Sealed Envelope [31]. No stratification was used. The generated allocation sequence contained 24 allocations, which were placed in sequentially numbered sealed envelopes and handed over to the study psychologist. During the enrolment session, the study psychologist opened the next envelope in sequence and assigned the participant to the corresponding treatment. The study psychologist was not blinded to treatment allocation and also conducted the follow-up assessments. Participants were aware of their allocated intervention.

### Intervention and control

#### Intervention group

Participants in the RNT-ACT group received two 60-min face-to-face sessions with at least 1 week between the sessions. The treatment protocol included four generic audio recordings corresponding to exercises introduced during the sessions. One recording was provided after the first session and the remaining three after the second session, with instructions to listen to one recording per day. The generic recordings contained no participant-specific information and were initially made available via SoundCloud [32] and later through the project website hosted by Lund University [33]. In accordance with the treatment protocol, participants could alternatively record the corresponding exercises during their own session using their personal phone for individual use.

The intervention was delivered by the study psychologist, who had been involved in the Swedish translation and adaptation of the RNT-ACT protocol. There is no formal certification process for RNT-ACT. Protocol adherence was supported by review of recorded sessions by a psychologist familiar with the protocol (A.L.) using the published treatment guide [15].

The first session introduced a functional analysis of RNT based on the protocol’s functional contextual and relational frame theory conceptualisation, followed by experiential ACT exercises adapted to interrupt repetitive negative thinking. The second session focused primarily on consolidating and maintaining the new response pattern through additional experiential exercises. A detailed description of the protocol is available in the original RNT-ACT publication [14]. The 1-month interval between the end of the face-to-face sessions and the main follow-up allowed participants time to continue practising with the audio recordings.

#### Control group

Participants randomised to iCBT received access to a suitable program from the national internet CBT platform (see [34] for a current list). Each program consists of approximately eight self-help modules, completed with therapist support each week. The iCBT program was selected based on the M.I.N.I. assessment together with the clinical assessment. For example, a patient with depression could be assigned a program targeting depression, with therapy content focused on behavioural activation or self-compassion. Modules include evidence-based CBT interventions such as psychoeducation, exposure, and relapse prevention, organised similarly to structured face-to-face CBT. ACT elements may be present, but no program was fully ACT-based. Therapist contact was available throughout for support and motivation.

#### Follow-up

At the main follow-up, scheduled approximately 1 month after completion of the allocated intervention, all study participants were offered a research follow-up session with the study psychologist and received a follow-up form in advance containing the secondary assessment scales and satisfaction questionnaires. The M.I.N.I. was administered during the follow-up session. The session was not part of the allocated intervention; however, participants could discuss questions and request subsequent treatment, including the alternative treatment arm. Three months after the follow-up session, a follow-up form was sent via secure communication.

Table 1 provides an overview of how the different measures are distributed, following the SPIRIT 2025 format [35]. In accordance with the RNT-ACT protocol, the second session could be scheduled 1–11 weeks after the first session [14]. Within this interval, the timing of the second session was determined by appointment availability in routine clinical practice.

**Table 1:** Participant timeline: schedule of enrolment, interventions, and assessments in SPIRIT format

Timepoint	Study period				
	Enrolment		Intervention period	Main follow-up	Three-month follow-up
	t-1	t0	t1	t2	t3
Enrolment					
Initial eligibility screening	X				
Informed consent		X			
Eligibility assessment		X			
Baseline assessment	X	X			
Randomisation		X			
Intervention/comparator*					
RNT-ACT			X		
iCBT			X		
Assessments					
Baseline assessment	M.I.N.I. 7.0.1 SMBQ/KEDS Blood test DASS-21 >20				
Primary feasibility outcome measures		Recruitment rate		Intervention non-completion CSQ-8	
Secondary outcome measures	DASS-21 RRS-BR SAAQ VQ CFQ-7			DASS-21 RRS-BR SAAQ VQ CFQ-7	DASS-21 RRS-BR SAAQ VQ CFQ-7

^*^RNT-ACT consisted of two 60-min sessions, with the second session scheduled 1–11 weeks after the first. iCBT consisted of approximately eight self-help modules, typically completed with therapist support over approximately 7–8 weeks. Follow-up assessments were not part of the allocated interventions

### Data collection and analysis

Data were collected via REDCap, a secure web-based platform hosted by Lund University [36, 37]. Participants completed questionnaires via secure communication [38] and received REDCap links. Video recordings of RNT-ACT sessions containing sensitive content were stored in an encrypted, GDPR-compliant environment with two-factor authentication. Analyses were descriptive and exploratory: means, standard deviations, and proportions were calculated. Analyses were based on available observations at each assessment point, and no imputation of missing data was performed. Consequently, the participants contributing data differed across assessment points. Missingness was considered potentially informative, particularly in the iCBT group, where follow-up data were available only from participants who remained active in the intervention. Individual change in DASS-21 total scores was assessed using the Reliable Change Index (RCI) according to Jacobson and Truax [39]. The calculation used the pooled baseline standard deviation of the present sample (SD = 10.28) and a reliability coefficient of *α* = 0.90, previously reported for the DASS-21 composite score in a primary care sample [40]. Reliable change was defined as RCI > 1.96, corresponding to a change of more than 9.01 points on the DASS-21 total score. All analyses were conducted in RStudio [41, 42].

### Changes to the protocol

The original trial registration covered both the pilot study and the planned definitive RCT and did not clearly distinguish between them. For the present pilot, recruitment, intervention non-completion, and patient satisfaction were treated as primary feasibility outcomes, while symptom and process measures were secondary outcomes. Before recruitment, WAI, BSRI, and AFQ-Y8 were removed to reduce assessment burden, AFQ-Y8 was replaced by the VQ, and the DASS-21 eligibility criterion was changed from ≥25 to >20. During recruitment, SMBQ-6 was replaced by KEDS on September 5, 2024, as updated guidance made KEDS more clinically useful for assessing the upper exhaustion threshold used for eligibility [22]. Of 37 individuals screened, 25 were assessed using SMBQ-6 and 12 using KEDS. A retrospective review indicated that this change did not affect the eligibility status of any randomised participant. A 60% recruitment rate criterion among eligible individuals was introduced during manuscript preparation after data collection and was not an a priori feasibility criterion.

## Results

**Fig. 1:**
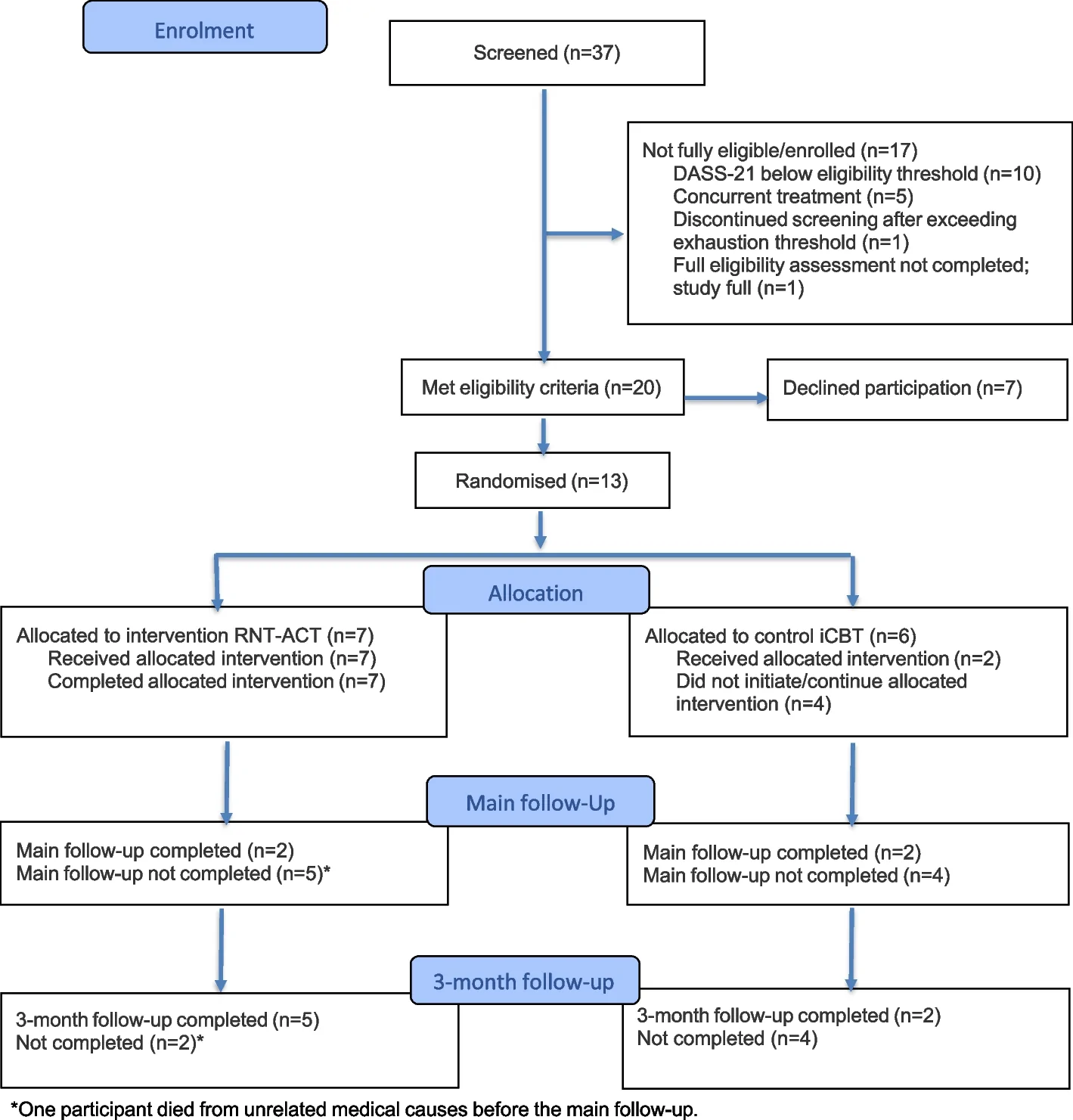
CONSORT flow diagram of participant flow

Over 9 months, from February to November 2024, 37 individuals were screened. Twenty met the eligibility criteria, of whom 13 consented to participate and were randomised (7 to RNT-ACT and 6 to iCBT), while seven declined participation. Of those not randomised, 10 had a DASS-21 score below the eligibility threshold, five were receiving concurrent psychological or psychiatric treatment, one discontinued screening after exceeding the exhaustion threshold, and one did not complete the full eligibility assessment because the recruitment target had been reached (Fig. 1). One participant did not complete the baseline assessment. No adverse events related to the study interventions were identified. One RNT-ACT participant died from unrelated medical causes between the intervention and the main follow-up. The event was considered unrelated to the study intervention. Three RNT-ACT participants missed the main follow-up but completed the 3-month follow-up. The median interval between the two RNT-ACT sessions was 21 days (range 7–71 days; mean 24.1 days). Participant flow is presented in Fig. 1.

The RNT-ACT group had 4/7 women, while the iCBT group had 2/6 women. Based on the M.I.N.I. assessment and clinical assessment, participants were categorised into six diagnostic groups. In the RNT-ACT group, three had a depressive diagnosis, three had an anxiety diagnosis, and one had a stress diagnosis. In the iCBT group, two had a depressive diagnosis, two had a social anxiety diagnosis, and one had a stress diagnosis. One in the iCBT group had a primary diagnosis of alcohol use disorder (Table 2).

**Table 2:** Patient data collected at enrolment

	RNT-ACT (*N* = 7)	iCBT (*N* = 6)	Overall (*N* = 13)
Age			
Mean (SD)	49.9 (8.04)	46.6 (4.42)	48.3 (6.59)
Median [min, max]	52.3 [34.4, 58.2]	45.5 [42.1, 53.6]	49.6 [34.4, 58.2]
Gender			
Women	4 (57.1%)	2 (33.3%)	6 (46.2%)
Men	3 (42.9%)	4 (66.7%)	7 (53.8%)
Diagnosis			
Alcohol use disorder F10	0 (0%)	1 (16.7%)	1 (7.7%)
Depression F32	2 (28.6%)	1 (16.7%)	3 (23.1%)
Major depressive disorder, recurrent F33	1 (14.3%)	1 (16.7%)	2 (15.4%)
Other (adjustment disorder F43.2)	1 (14.3%)	1 (16.7%)	2 (15.4%)
Social anxiety disorder F40.10	0 (0%)	2 (33.3%)	2 (15.4%)
Generalised anxiety disorder F41.1	3 (42.9%)	0 (0%)	3 (23.1%)
DASS-21 total score baseline			
Mean (SD)	29.3 (5.15)	33.6 (15.4)	31.1 (10.3)
Median [min, max]	28.0 [21.0, 37.0]	30.0 [19.0, 57.0]	29.0 [19.0, 57.0]
Missing	0 (0%)	1 (16.7%)	1 (7.7%)
RRS-BR total score baseline			
Mean (SD)	26.0 (6.43)	23.6 (9.63)	25.0 (7.60)
Median [min, max]	27.0 [17.0, 36.0]	25.0 [13.0, 36.0]	26.0 [13.0, 36.0]
Missing	0 (0%)	1 (16.7%)	1 (7.7%)
SAAQ total score baseline			
Mean (SD)	19.1 (3.98)	23.8 (10.1)	21.1 (7.19)
Median [min, max]	19.0 [14.0, 26.0]	21.0 [14.0, 35.0]	20.0 [14.0, 35.0]
Missing	0 (0%)	1 (16.7%)	1 (7.7%)
VQ Progress total score baseline			
Mean (SD)	16.1 (4.98)	16.8 (6.69)	16.4 (5.47)
Median [min, max]	13.0 [12.0, 25.0]	15.0 [10.0, 28.0]	15.0 [10.0, 28.0]
Missing	0 (0%)	1 (16.7%)	1 (7.7%)
VQ Obstruction total score baseline			
Mean (SD)	21.4 (2.44)	22.8 (7.89)	22.0 (5.13)
Median [min, max]	22.0 [18.0, 24.0]	25.0 [11.0, 30.0]	22.5 [11.0, 30.0]
Missing	0 (0%)	1 (16.7%)	1 (7.7%)
CFQ-7 total score baseline			
Mean (SD)	34.3 (6.95)	31.6 (12.5)	33.2 (9.24)
Median [min, max]	33.0 [27.0, 45.0]	36.0 [17.0, 46.0]	34.5 [17.0, 46.0]
Missing	0 (0%)	1 (16.7%)	1 (7.7%)

*DASS-21* The Depression, Anxiety and Stress Scale 21 items, *RRS-BR* Ruminative Responses Scale—Brooding and Reflection, *SAAQ* Swedish Acceptance and Action Questionnaire, *VQ Progress* Progress subscale on Values Questionnaire, *VQ Obstruction* Obstruction subscale on Values Questionnaire, *CFQ-7* Cognitive Fusion Questionnaire 7 items

### Primary outcomes

Of the 37 individuals screened, 13 were enrolled and randomised, resulting in a 35% overall recruitment rate and a 65% recruitment rate among eligible patients. All seven participants allocated to RNT-ACT completed the two-session intervention (intervention non-completion: 0%), whereas two of six participants allocated to iCBT completed the intervention (intervention non-completion: 67%).

Completion of the research follow-up assessments was considered separately from intervention completion. In the RNT-ACT group, two of seven participants completed the main follow-up as scheduled. Five of seven participants completed the 3-month follow-up, including three who had not completed the main follow-up. In the iCBT group, two of six participants completed both the main and 3-month follow-up assessments.

Some participants received additional treatment contacts after the allocated intervention as part of subsequent clinical care; these were not considered part of the allocated intervention or the research follow-up assessments. Treatment satisfaction, measured using the CSQ-8, had a mean score of 27.8 (SD 3.7; *n* = 5) in the RNT-ACT group and 23.0 (SD 0.0; *n* = 2) in the iCBT group. For three RNT-ACT participants who did not complete the main follow-up, CSQ-8 data were collected at the 3-month follow-up. Table 3 provides further details on intervention completion, additional treatment contacts, and therapist-reported time spent on the intervention. Research assessments and follow-up sessions were excluded from the intervention time estimates.

**Table 3:** Intervention completion, research follow-up, and additional clinical contacts by participant

Allocation	Study-ID	Intervention completion	Research follow-up/additional clinical contacts	Therapist-reported treatment time including additional sessions*
RNT-ACT	I1	Completed	Did not complete the main follow-up; completed the 3-month follow-up. Received additional medical treatment between the main follow-up and 3-month follow-up	120 min
RNT-ACT	I2	Completed	Lost to research follow-up after intervention. No additional treatment contacts	120 min
RNT-ACT	I3	Completed	Did not complete the main follow-up; completed the 3-month follow-up. Received one additional clinical contact (45 min)	165 min
RNT-ACT	I4	Completed	Did not complete the main follow-up; completed the 3-month follow-up. Started additional treatment contact (asked for referral)	120 min
RNT-ACT	I5	Completed	Completed both research follow-ups. Received two additional treatment sessions between the main and 3-month follow-ups	210 min
RNT-ACT	I6	Completed	Completed both research follow-ups. Received two additional treatment sessions during the follow-up period	210 min
RNT-ACT	I7	Completed	Deceased from unrelated medical causes before the main follow-up	120 min
iCBT	C1	Did not initiate	Did not complete the research follow-ups	NA
iCBT	C2	Completed	Completed both research follow-ups. Reported insufficient treatment effect and subsequently received RNT-ACT after the main follow-up	80 min + 120 min subsequent RNT-ACT
iCBT	C3	Completed	Completed both research follow-ups. No additional treatment sessions	147 min
iCBT	C4	Did not complete	Withdrew from the study and did not complete the research follow-ups	NA
iCBT	C5	Did not complete	Inactive in the iCBT program; did not complete the research follow-ups	NA
iCBT	C6	Did not complete	Inactive in the iCBT program; did not complete the research follow-ups	NA

^*^Treatment time included treatment-related clinical administration but excluded research-related administration and research assessments. NA indicates that treatment time was not estimated

### Secondary outcomes

Secondary outcomes were analysed descriptively using available observations. Mean DASS-21 scores were 29.29 (SD 5.15; *n* = 7) at baseline, 15.00 (SD 1.41; *n* = 2) at the main follow-up, and 12.20 (SD 9.65; *n* = 5) at the 3-month follow-up in the RNT-ACT group. Corresponding scores in the iCBT group were 33.60 (SD 15.40; *n* = 5), 23.50 (SD 2.12; *n* = 2), and 18.50 (SD 4.95; *n* = 2), respectively. Because different participants contributed data at different assessment points, these values should not be interpreted as within-person trajectories or between-group treatment effects. Reliable improvement in DASS-21 scores was observed in 2/2 RNT-ACT participants and 1/2 iCBT participants with available data at the main follow-up, and in 4/5 and 1/2 participants, respectively, at the 3-month follow-up. Given the small and changing samples, these findings are descriptive only. Results for the remaining secondary outcome measures are reported in Additional files 2 and 3.

## Discussion

### Main findings

This pilot study identified important feasibility considerations for a future trial of RNT-ACT versus iCBT in Swedish primary care. Recruitment among eligible patients was 65%, although overall recruitment was low (35%) and slower than anticipated. All participants allocated to RNT-ACT completed the two-session intervention, whereas four of six participants allocated to iCBT did not complete treatment. Satisfaction among participants providing follow-up data was acceptable, but completion of research follow-up was suboptimal. Although symptom improvement was observed among participants with available data, the small sample and substantial missing data preclude conclusions regarding comparative treatment effects.

There are two possible design-related explanations for the low completion of the main research follow-up in the RNT-ACT group. One is the number of questionnaires to be completed at follow-up. Another is the time interval between the end of treatment and the main follow-up. The 1-month follow-up point was based on previous RNT-ACT research and the treatment protocol [14, 43]. However, this pilot does not establish that 1 month is the optimal follow-up interval. Future studies should therefore investigate how outcome assessment can be made both clinically meaningful and feasible within routine primary care.

#### Secondary measurements

Interpretation of the secondary outcomes is limited by the small sample and substantial missing data. The DASS-21 was useful as a measure of global distress, but this pilot does not provide sufficient evidence to determine its role as a primary outcome or to establish an optimal eligibility threshold for a future trial. The secondary outcome data should not be interpreted as evidence of comparative treatment efficacy, as this pilot was not designed or powered to test treatment effects, and the analyses were based on small and changing samples with substantial missing data.

### Strengths and limitations

A strength of this pilot study is that it evaluated RNT-ACT within routine primary care and compared it with an established treatment available in the same setting. However, the small sample size, low overall recruitment, substantial intervention non-completion in the iCBT group, and missing follow-up data substantially limit interpretation of between-group differences. In particular, the participants contributing outcome data differed across assessment points, and missingness was likely informative. Patient satisfaction data were available only from participants who provided follow-up data and therefore may not represent participants who did not initiate or complete the intervention. Consequently, the secondary outcome findings should be interpreted as descriptive and cannot provide estimates of comparative treatment effects. The small sample also results in considerable uncertainty in the feasibility estimates; proportions such as intervention non-completion should therefore not be interpreted as stable estimates of expected rates in a larger population.

All RNT-ACT sessions were delivered by a single psychologist, who was also involved in the development and Swedish adaptation of the intervention. This ensured consistency in treatment delivery but limits the extent to which intervention feasibility can be generalised to other therapists and routine clinical delivery. The feasibility of therapist training and delivery by clinicians with varying experience therefore requires further investigation.

### Comparison with other studies

Intervention non-completion in the iCBT group was consistent with previous reports of non-initiation and early discontinuation of iCBT in primary care [8, 44]. However, the small sample precludes conclusions about the reasons for non-completion or the suitability of iCBT as a comparator. Previous qualitative research suggests that treatment format and preferences for face-to-face therapy may influence engagement with iCBT, although differences in treatment setting and therapist support limit direct comparison with the present study [45].

Recruitment for RCTs in primary care can be challenging, particularly for depression [46]. In the present study, recruitment targeted patients seeking care at PHCCs and described treatment for depression, anxiety, and stress. In contrast, previous RNT-ACT studies recruited through social media using messages focused more directly on overthinking and repetitive negative thinking [47]. These differences in setting and recruitment framing may have contributed to the slower recruitment observed in the present study. The prolonged recruitment period also required continued involvement of primary care staff, highlighting the need to consider the resource demands of recruitment when designing future studies in routine primary care. Patient preferences regarding treatment duration, treatment format, and continuity with a clinician may also influence recruitment and engagement, but these factors were not assessed in the present study. Qualitative investigation of patient and therapist experiences may help clarify their relevance when adapting brief transdiagnostic interventions to primary care.

### Implications for further research

The pilot study identified several issues that should be addressed before considering a full-scale RCT. In particular, further work is needed to understand recruitment, treatment engagement, outcome assessment, and how the intervention can be implemented within routine primary care. The burden and timing of research assessments should be reconsidered, with particular attention to outcome assessment that is both clinically meaningful and feasible in this setting.

The findings also indicate a need to further investigate how RNT-ACT is understood and delivered in clinical practice. Qualitative investigation of patient and therapist experiences may provide important information about recruitment, treatment engagement, therapist training, and potential adaptations of the intervention and study procedures. These questions should be addressed before determining the design and eligibility criteria of any future definitive trial.

## Conclusion

This pilot study identified important feasibility challenges related to recruitment, treatment engagement, and research follow-up in a trial comparing RNT-ACT with iCBT in Swedish primary care. The small sample and substantial intervention non-completion in the iCBT group limit between-group comparisons. Further investigation of recruitment, treatment engagement, and patient and therapist experiences is needed before determining whether and how to proceed to a full-scale RCT.

## Supplementary Information

Additional file 1: Scales used in the pilot trial.

Additional file 2: Secondary outcome measures descriptive statistics.

Additional file 3: Reliable change in DASS-21 scores.

Additional file 4: CONSORT Extension for Pilot and Feasibility Trials checklist.

## Data Availability

Data will be made available on a reasonable request to the corresponding author.

## Abbreviations

ACT: Acceptance and commitment therapy
CPF: Centre for Primary Care Research, in Swedish “Centrum för Primärvårdsforskning”, Skåne, Sweden
iCBT: Internet-delivered cognitive behavioural therapy
CBT: Cognitive behavioural therapy
GAD: Generalised anxiety disorder
PHCC: Primary Health Care Centre
RNT: Repetitive negative thinking
RNT-ACT: An acceptance and commitment therapy protocol focused on repetitive negative thinking
SALAR: Swedish Association of Local Authorities and Regions, in Swedish “SKR” (Sveriges Kommuner och Regioner)
SiBER: The Swedish National Quality Register for internet-based psychological treatment
SoB: Stöd och Behandling (support and treatment)
