# Supplementary material for: Feasibility of acceptance and commitment therapy for repetitive negative thinking versus internet-based CBT: a pilot randomised controlled trial in Swedish primary care among patients with anxiety, depression and stress
Source: Pilot Feasibility Stud. 2026 Oct 31;12:155. doi: 10.1186/s40814-026-01951-2 (PMC13723136; doi:10.1186/s40814-026-01951-2)
Supplement: Supplementary file 1 [file 40814_2026_1951_MOESM1_ESM.docx]

# Additional file 1 – Scales used in the pilot study

### Mini International Neuropsychiatric Interview (M.I.N.I 7.0.1)

M.I.N.I 7.0.1 is a structured interview guide for the classification of common psychiatric diagnosis (1, 2). It is an established procedure in research and in many clinical settings. It is mostly based on Yes/No questions which emulate the DSM-5 criteria for the diagnosis.

### SHIROM-MELAMED BURNOUT MEASURE-6 (SMBQ-6)

SMBQ-6 is a six-item measure of exhaustion/fatigue with a total score range of 6-42 (3-5). It was replaced in the mid-study by the KEDS measurement.

### KAROLINSKA EXHAUSTION DISORDER SCALE (KEDS)

KEDS is a nine-item scale measuring stress-induced exhaustion disorder with a total score range of 0-54, where higher scores indicating more exhaustion symtoms. During the pilot study norms were presented where/exhaustion could be influencing the ability to receive therapy (6). Therefore, KEDS replaced SMBQ, and the exclusion criteria were set to 30 or over.

### Client Satisfaction Scale (CSQ-8).

The CSQ-8 is an established measure of the client's satisfaction with the treatment based on 8 items (7). The items are rated at the termination of therapy and scores from 1 to 4 with a total between 8-32 where a higher number indicates higher satisfaction with therapy.

### The Depression, Anxiety and Stress Scale-21 (DASS-21)

The Depression, Anxiety and Stress Scale-21 (DASS-21) is a widely used measure of psychological symptoms and distress (8). It is a self-report measure assessing symptoms of depression, anxiety, and stress during the previous week. Each subscale consists of seven items, and the combined scale can be used as a measure of overall distress. The 21 items are rated on a 0–3 Likert scale, giving a total score range of 0–63. The DASS-21 >20 criterion was not intended as a diagnostic cut-off. The 20/21 threshold was calculated using the Jacobson and Truax method (9) based on Swedish DASS-21 data reported by Alfonsson et al. (8) and was used as a symptom-related eligibility threshold in the present study. For the Reliable Change Index calculation, a Cronbach’s alpha of 0.90, previously reported for the DASS-21 composite score in a primary care sample (10), was used.

### Ruminative Responses Scale – Brooding and Reflection (RRS-BR)

RRS-BR is an internationally established measurement of the tendency to ruminate (11). It is a self-report scale where the ten items are graded on frequency on two subscales, brooding and self-reflection. Total score range 10-40, subscales have score range 5-20 each. Higher values indicate a higher tendency to ruminate. Cronbach’s alpha on the one-dimensional scale is .87.

### Swedish Acceptance and Action Questionnaire (SAAQ)

The SAAQ is the Swedish translation of the internationally widely used Acceptance and Action Questionnaire (AAQ-II) (12). The scale consists of 6 items ranged from 1 to 7 with a higher value indicates a higher frequency of alignment with the statement and a higher degree of the construct psychological inflexibility. Total score range is 6-42, Cronbach’s α is .85

### Values Questionnaire (VQ)

Values Questionnaire is a 10-items self-assessment for valued living, to what extent a person lives a life that they value (13). The Swedish translation of the scale consists of 10 items scored 1-7 on a seven-point Likert scale with two subscales, progress and obstruction. Each subscale has a score range of 5-35, Cronbach's Alpha was.83 is Progress and 0.76 for Obstruction. Scores indicative of higher valued living are high scores on the Progress scale (3,4,5,7,9) and low scores on the obstruction scale (1,2,6,8,10).

### Cognitive Fusion Questionnaire – Swedish (CFQ-7)

The Cognitive Fusion Questionnaire is a seven items Likert self-report scale ranging from 1 to 7 with a total score range of 7-49 (14). A higher value indicates a higher degree of the ACT process known as “fusion” which can be described as a potentially pathological psychological process where the person sees the thoughts and learning history as true on the expense of real-world contingencies. The scale has a one-dimensional structure and Cronbach’s alpha is set to (α=.88-.93). In addition, the Spanish version of CFQ was found sensitive to an early version of the RNT-ACT protocol (15).

# References

1.	Sheehan DV, Lecrubier Y, Harnett Sheehan K, Janavs J, Weiller E, Keskiner A, et al. The validity of the Mini International Neuropsychiatric Interview (MINI) according to the SCID-P and its reliability. European Psychiatry. 1997;12(5):232-41.

2.	Sheehan DV, Lecrubier Y, Sheehan KH, Amorim P, Janavs J, Weiller E, et al. The Mini-International Neuropsychiatric Interview (M.I.N.I.): the development and validation of a structured diagnostic psychiatric interview for DSM-IV and ICD-10. J Clin Psychiatry. 1998;59 Suppl 20:22-33;quiz 4-57.

3.	Almén N, Jansson B. The reliability and factorial validity of different versions of the Shirom-Melamed Burnout Measure/Questionnaire and normative data for a general Swedish sample. International Journal of Stress Management. 2021;28(4):314.

4.	Almén N, Jansson B. Validation of the new brief 6-item version of the Shirom-Melamed Burnout Measure. Cogent Psychology. 2023;10(1):2258476.

5.	Sundström A, Söderholm A, Nordin M, Nordin S. Construct validation and normative data for different versions of the Shirom‐Melamed burnout questionnaire/measure in a Swedish population sample. Stress and Health. 2023;39(3):499-515.

6.	Åsberg M. Utmattningssyndrom. Stockholm: Gothia kompetens; 2024.

7.	Attkisson CC, Zwick R. The client satisfaction questionnaire. Psychometric properties and correlations with service utilization and psychotherapy outcome. Eval Program Plann. 1982;5(3):233-7.

8.	Alfonsson S, Wallin E, Maathz P. Factor structure and validity of the Depression, Anxiety and Stress Scale-21 in Swedish translation. J Psychiatr Ment Health Nurs. 2017;24(2-3):154-62.

9.	Jacobson NS, Truax P. Clinical significance: a statistical approach to defining meaningful change in psychotherapy research. J Consult Clin Psychol. 1991;59(1):12-9.

10.	Berger T, Urech A, Krieger T, Stolz T, Schulz A, Vincent A, et al. Effects of a transdiagnostic unguided Internet intervention ('velibra') for anxiety disorders in primary care: results of a randomized controlled trial. Psychological Medicine. 2017;47(1):67-80.

11.	Cronwall E. Psykometrisk evaluering av RRS-BR och BSRI i svensk översättning2019 2019.

12.	Lundgren T, Parling T. Swedish Acceptance and Action Questionnaire (SAAQ): a psychometric evaluation. Cognitive Behaviour Therapy. 2017;46(4):315-26.

13.	Rickardsson J, Zetterqvist V, Kemani MK, Holmström L, Andersson E, Wicksell RK. Assessing values – Psychometric properties of the Swedish version of the Valuing Questionnaire in adults with chronic pain. Journal of Contextual Behavioral Science. 2019;14:40-9.

14.	Gillanders DT, Bolderston H, Bond FW, Dempster M, Flaxman PE, Campbell L, et al. The Development and Initial Validation of the Cognitive Fusion Questionnaire. Behavior Therapy. 2014;45(1):83-101.

15.	Ruiz FJ, Suárez-Falcón JC, Riaño-Hernández D, Gillanders D. Psychometric properties of the Cognitive Fusion Questionnaire in Colombia. Revista Latinoamericana de Psicología. 2017;49(1):80-7.
