# Supplementary material for: Feasibility of acceptance and commitment therapy for repetitive negative thinking versus internet-based CBT: a pilot randomised controlled trial in Swedish primary care among patients with anxiety, depression and stress
Source: Pilot Feasibility Stud. 2026 Oct 31;12:155. doi: 10.1186/s40814-026-01951-2 (PMC13723136; doi:10.1186/s40814-026-01951-2)
Supplement: Supplementary file 2 [file 40814_2026_1951_MOESM2_ESM.docx]

Additional file 2 - Secondary outcome measures: descriptive statistics

|  | **RNT-ACT** | | | | | **iCBT** | | | | |
| --- | --- | --- | --- | --- | --- | --- | --- | --- | --- | --- |
| **Variable** | **N** | **Min** | **Max** | **Mean** | **SD** | **N** | **Min** | **Max** | **Mean** | **SD** |
| DASS-21 Baseline | 7 | 21 | 37 | 29.29 | 5.15 | 5 | 19 | 57 | 33.6 | 15.40 |
| DASS-21 Main follow-up | 2 | 14 | 16 | 15.00 | 1.41 | 2 | 22 | 25 | 23.5 | 2.12 |
| DASS-21 3 months | 5 | 2 | 26 | 12.20 | 9.65 | 2 | 15 | 22 | 18.5 | 4.95 |
| RRS-BR Baseline | 7 | 17 | 36 | 26.00 | 6.43 | 5 | 13 | 36 | 23.6 | 9.63 |
| RRS-BR Main follow-up | 2 | 19 | 20 | 19.50 | 0.71 | 2 | 19 | 19 | 19.0 | 0.00 |
| RRS-BR 3 months | 5 | 11 | 24 | 17.80 | 5.26 | 2 | 14 | 20 | 17.0 | 4.24 |
| SAAQ Baseline | 7 | 14 | 26 | 19.14 | 3.98 | 5 | 14 | 35 | 23.8 | 10.13 |
| SAAQ Main follow-up | 2 | 11 | 19 | 15.00 | 5.66 | 2 | 16 | 16 | 16.0 | 0.00 |
| SAAQ 3 months | 5 | 7 | 20 | 13.80 | 4.66 | 2 | 17 | 21 | 19.0 | 2.83 |
| VQ Progress Baseline | 7 | 12 | 25 | 16.14 | 4.98 | 5 | 10 | 28 | 16.8 | 6.69 |
| VQ Progress Main follow-up | 2 | 15 | 17 | 16.00 | 1.41 | 2 | 16 | 22 | 19.0 | 4.24 |
| VQ Progress 3 months | 5 | 15 | 31 | 23.40 | 7.37 | 2 | 13 | 24 | 18.5 | 7.78 |
| VQ Obstruction Baseline | 7 | 18 | 24 | 21.43 | 2.44 | 5 | 11 | 30 | 22.8 | 7.89 |
| VQ Obstruction Main follow-up | 2 | 12 | 16 | 14.00 | 2.83 | 2 | 16 | 19 | 17.5 | 2.12 |
| VQ Obstruction 3 months | 5 | 8 | 19 | 13.40 | 5.50 | 2 | 12 | 23 | 17.5 | 7.78 |
| CFQ-7 Baseline | 7 | 27 | 45 | 34.29 | 6.95 | 5 | 17 | 46 | 31.6 | 12.54 |
| CFQ-7 Main follow-up | 2 | 17 | 26 | 21.50 | 6.36 | 2 | 19 | 20 | 19.5 | 0.71 |
| CFQ-7 3 months | 5 | 9 | 27 | 20.80 | 7.46 | 2 | 20 | 30 | 25.0 | 7.07 |

DASS-21 = The Depression, Anxiety and Stress Scale 21 items, RRS-BR = Ruminative Responses Scale – Brooding and Reflection, SAAQ = Swedish Acceptance and Action Questionnaire, VQ Progress = Progress subscale on Values Questionnaire, VQ Obstruction = Obstruction subscale on Values Questionnaire, CFQ-7 = Cognitive Fusion Questionnaire 7 items.
