# Supplementary material for: Feasibility of acceptance and commitment therapy for repetitive negative thinking versus internet-based CBT: a pilot randomised controlled trial in Swedish primary care among patients with anxiety, depression and stress
Source: Pilot Feasibility Stud. 2026 Oct 31;12:155. doi: 10.1186/s40814-026-01951-2 (PMC13723136; doi:10.1186/s40814-026-01951-2)
Supplement: Supplementary file 3 [file 40814_2026_1951_MOESM3_ESM.docx]

Additional file 3. Proportion of participants with Reliable Change on DASS-21 per group

| **Timepoint** | **Allocation** | **N** | **N with reliable improvement** |
| --- | --- | --- | --- |
| Baseline–Main | RNT-ACT | 2 | 2 |
| Baseline–Main | iCBT | 2 | 1 |
| Baseline–3 month | RNT-ACT | 5 | 4 |
| Baseline–3 month | iCBT | 2 | 1 |

DASS-21 = The Depression, Anxiety and Stress Scale 21 items

o
